# Risk Factors for Opioid Use Disorders in Adult Postsurgical Patients

**DOI:** 10.7759/cureus.2611

**Published:** 2018-05-11

**Authors:** Kimberly M Burcher, Andrey Suprun, Arron Smith

**Affiliations:** 1 Medical Student, University of Central Florida College of Medicine, Orlando, USA

**Keywords:** opioid, use, abuse, opioid use disorder, misuse, dependence, risk factors, patient derived, narcotic

## Abstract

The use of opioids in the treatment of chronic pain is one of the most controversial topics in medicine today. Many studies have proposed that the postoperative period is a vulnerable time for patients at risk for developing an opioid use disorder. Many patients are prescribed opioids for management of their postsurgical pain and continue using them for prolonged amounts of time following their surgeries. Some populations are more likely to develop an opioid use disorder following exposure to opioid medications than others. In this review, the authors discuss the patient-level risk factors for the abuse of these drugs in postsurgical patients.

## Introduction and background

The use of opioids in the treatment of chronic pain is one of the most controversial topics in medicine today [1]. Over the past 10 years, addiction to opioid analgesics has emerged as a major issue, recently reaching epidemic status. In 2015, 20.5 million Americans aged 12 years and older suffered a substance abuse disorder. Two million of these Americans had a disorder involving prescription pain relievers (including opioid medications) and 591,000 had a disorder involving heroin [2] (for which prescribed opioids often serve as a “gateway”). Some populations, such as those with a history of substance abuse, have been shown to have an even higher incidence of abuse with 60 to 70% of patients prescribed opioids for treatment of legitimate pain later utilizing drugs of the same class for recreation [3].

Increases in opioid abuse can be attributed at least in part to changes in medication prescribing habits, changes in drug formulations, increased ease of access to drugs illegally (including over the internet), and an overall massive increase in the number of opioid prescriptions filled [4]. While opioid analgesic use in acute pain management seems benign, it often results in long-term use of opioids for pain management, which is associated with significant burden and addiction [4, 5]. Furthermore, the scant evidence for the long-term efficacy of opioids does not support their widespread use in the management of chronic pain and raises concerns for patients’ greater physical and psychosocial problems [6].

Every day in the United States, 650,000 opioid prescriptions are filled with a significant portion of this utilization being due to inappropriate prescription by medical professionals [3]. In 2015, 97.5 million patients used prescription pain relievers, representing more than one third (36.4%) of the population older than 12 years of age. In the same year, 12.5 million misused pain medications, of which 2.1 million were new misusers of pain medications including adolescents ages 12 through 17 and adults ages 18 and older. The sale of opioids has increased in particular for the management of non-cancer pain, [7, 8] and many experts have postulated that there is a correlation between this finding and the increase in opioid overdoses and deaths [9, 10]. The current Centers for Disease Control (CDC) recommendation is that all physicians exercise caution while prescribing opioid medications for chronic non-cancer pain that is not palliative or related to end of life care [11].

Today, approximately $55.7 billion in annual health and societal costs are related to prescription opioid abuse and misuse, with $20 billion in spending due to related emergency department visits and hospitalizations for overdoses, cellulitis, abscess formation, bacteremia, sepsis, endocarditis, and osteomyelitis [12, 13]. The opioid-related death rate has increased by 285% in data comparing incidence between 2002 and to incidence between 2011 and 2014. In 2015, approximately 58.3 million people used hydrocodone products while only 828,000 people used heroin [14].

An inherent difficulty in the study of opioid abuse and misuse is the seeming interchangeability of these and related terms [1]. Terms such as “opioid abuse,” “opioid misuse,” and “opioid dependence” make up a group of distinct disorders which have been described in detail and impeccably defined. Therefore each relevant term is defined in Table 1 using definitions adapted from "Prescription Opioid Abuse in Chronic Pain: An Updated Review of Opioid Abuse Predictors and Strategies to Curb Opioid Abuse: Part 1" [3]. In this paper, each of these opioid-related conditions will be referred to as opioid use disorders (OUD).

**Table 1:** Definitions of Opioid Use Disorders

Concept	Definition
Substance use disorder	A group of intellectual, behavioral and physical symptoms that imply a patient has continued using a substance despite substantial substance-related complications. The diagnosis is based on a compulsive pattern of behaviors that can be attributed to the use of the substance.
Tolerance	A condition of adaptation described as distinct increases in dosage of a drug required to accomplish desired effect. May also be diagnosed in patients in whom repeat dosing results in lessening of one or more opioid effects over time.
Physical Dependence	A condition of adaptation demonstrated by patients who experience withdrawal syndromes elicited by abrupt cessation, dose reduction, lowered blood concentration of the drug, and/or administration of an antagonist.
Addiction	A chronic, neurobiologic disease with psychosocial, genetic, and environmental variables prompting its development. Addicted patients exemplify one or more of the following: failure to control drug use, habitual use, use without regard to the harm it causes, and craving. This term is considered outdated.
Aberrant drug-related behavior	A behavior that does not agree with the treatment plan made between a patient and his or her doctor.
Misuse	Use of a medication for nonmedical use, or for reasons other than prescribed. Misuse can be intentional or unintentional use of an opioid or other drug in a way inconsistent with medical guidelines or the law, including changes made to drug doses or the sharing of medications.
Abuse	Misuse with prominent consequences. Often defined as substance use with the purpose to adapt or control attitude or state of mind in a way that is not legal or damaging to oneself or others.
Diversion	The purposeful exchange of a controlled substance from legal distribution and dispensing channels into illegal channels.
Withdrawal	A health condition occurring when the concentration of a substance in the blood or tissue are lowered in patients who had previously maintained high concentrations as a result of extended and exorbitant use. The conditions described vary between drug classes and patient to patient.

The study of postsurgical pain management is sparse despite the fact that 80% of patients experience acute postsurgical pain [15, 16]. Many studies have proposed that the postoperative period is a vulnerable time for patients at risk of developing an OUD [17-19]. Due to the high number of patients prescribed opioids for the first time after surgery and the high proportion of patients who use opioids for a long duration postsurgically, it is likely that many patients who are exposed to opioids for the first-time following surgery develop an OUD. In 2017, Brummett et al. determined that general postsurgical patients were at increased risk for chronic opioid use [10]. Though current studies vary in their findings, about 1 in 30 patients exposed to opioids after major surgery continue to use them after three months [18].

With the number of patients sufferring OUDs on the rise, efforts have been made to identify patients at risk of developing OUD prior to opioid exposure. Many tools, (including the Opioid Risk Tool (ORT), the Screener and Opioid Assessment for Patients with Pain Version 1 (SOAPP-R), and Brief Risk Interview) have been developed as potential risk assessors for OUD. The CDC, however, is wary of the possible overestimation of the efficacy of existing OUD risk assessors’ ability to rule out the risks related to opioid prescriptions in individual patients. Furthermore, the CDC feels that the most important risk factor to be assessed before a prescription for opioids is given is a history of substance abuse [11]. According to CDC guidelines, currently existing risk stratification tools have not proven to be acceptably accurate. It has been speculated that each of the available options has failed to become the “gold standard” due to requirements for further validity testing, adjusting the weight of different risk factors with varying levels of importance, and some omission of risk factors between tools. Due to lack of agreement and accuracy of the tools, the CDC advocates that the risk of development of a substance abuse disorder be considered for all patients prescribed opioids rather than only for those with scores indicating increased risk on the available risk assessment tools [11].

While studies have made a distinction between the postsurgical and general population with regards to OUD risk, current research suggests that this may not be relevant. In research completed in 2016 by Sun et al., every surgical procedure included in the study (including total knee arthroplasty (TKA), total hip arthroplasty, laparoscopic cholecystectomy, open cholecystectomy, laparoscopic appendectomy, open appendectomy, cesarean delivery, functional endoscopic sinus surgery (FESS), cataract surgery, transurethral prostate resection (TURP), and simple mastectomy) was found to have an increased risk of chronic opioid use [20]. Other studies have found that postoperative opioid misuse cannot be accounted for with a statistically significant difference in incidence of patients undergoing minor versus major surgical procedures. Instead opioid abuse is associated with behavioral and pain disorders in conjunction with many patient-level risk factors for OUDs. These findings suggest that prolonged opioid use is not due to surgical pain but rather due to these same patient-level predictors unaffected by surgical status. The risk of OUD, regardless of the reason behind initial opioid exposure (surgical or otherwise), is much more correlated to patients’ risk factors than the reason for the exposure [10, 21]. Though status as a postsurgical patient increases likelihood of exposure to opioids, it does not alter the individual’s risk factors for OUD in comparison to the non-surgical population. Therefore, screening tools that have been validated for non-surgical patients can be utilized for surgical patients.

Creation of an effective screening tool will allow physicians and other medical professionals to appropriately handle the opioid crisis on an individual patient-to-patient-basis. The purpose of this review is to consolidate findings from several studies that explore the potential risks and predispositions which lead adult postsurgical patients to develop addiction to the opioids. The risk factors being reviewed in this paper are patient-centered. The authors will not discuss the effects of individual physician’s prescribing practices that may add to the patient’s risk. Six articles were reviewed and evaluated.

## Review

In 2010, a study completed by Edlund and colleagues ("Risks for Opioid Abuse and Dependence Among Recipients of Chronic Opioid Therapy: Results from the TROUP Study") was designed to estimate the risk factors for opioid abuse and dependence in patients on Continuous Opioid Therapy (COT) (defined as treatment duration over 90 days with multiple opioid prescription claims with no more than 32 days between opioid fill dates) enrolled in Arkansas Medicaid or registered in the HealthCore Integrated Research Database (a bank of health information from five commercial health plans based in the West, Mid-West, and South-East regions of the United States). The sample consisted of patients 18 years of age and older who were enrolled in these programs and on COT without diagnoses of cancer or residence in a nursing home. The study participants were not limited to the postsurgical population. The study identified 1,188 patients in the HealthCore sample (n=36,605) and 277 patients in the Arkansas Medicaid Sample (n=9,651) with a claims-based opioid abuse/dependence diagnosis [22].

This study noted that predictors of COT were almost the same in the two groups, but because the Medicaid sample was smaller, some risk factors were only significant in the HealthCore sample. The study found that younger patients (particularly those aged between 18 and 30 years) were at increased risk of OUD with the highest odds ratio noted when comparing the 18-30 year olds with 65 and over age group (OR 5.88, 9.08 in HealthCore and Arkansas respectively). Additionally, patients with back pain or headache diagnoses in the HealthCore sample were also statistically more likely to have opioid abuse or dependence claims. These variables, however, were a near miss for significance in the Arkansas Medicaid group at the *p*=.05 level. Other significant risk factors for OUD included mental health disorders and substance abuse, as well as the use of sedatives/hypnotics. Prescription controlled variables such as higher opioid dose, longer duration of opioid supply, and treatment plans containing certain types of opioids (particularly Schedule II long-acting opioids) also showed statistically significant increase in risk for COT [22].

The authors of this review believe the article "Risks for Opioid Abuse and Dependence Among Recipients of Chronic Opioid Therapy: Results from the TROUP Study" by Edlund et al. is of high clinical significance and chose to include it due to its large sample size, explicit explanation of procedures and careful consideration of inclusion/exclusion criteria. Though this study did not exclusively sample from a pool of postsurgical patients, its findings are representative of patients postsurgically. Previous research has stated that risk factors for OUD in these patients is related more to the patients’ individual risk factors than the surgery itself. For postsurgical patients, surgery serves merely as an initial exposure to opioids. OUD may set in for those at increased risk [10, 21]. This study is not without its limitations; it (like many others included in this review) was designed to be observational and the described statistics are associations not meant to be considered causal relationships. Additionally, though the sample size was large and covered a wide geography it may not be representative of the general population. Finally, this study failed to address the severity of pain patients experienced and the role of pain relief (or lack thereof) in their potential development of OUD.

In a second article, "A Pilot Cohort Study of the Determinants of Longitudinal Opioid Use After Surgery," Carroll et al. explored the role of preoperative psychological distress and history of substance abuse disorders in the development of postoperative OUD. This was a prospective cohort study that enrolled 109 patients undergoing one of the following surgeries: mastectomy, lumpectomy, thoracotomy, TKA, or total hip replacement. Patients were excluded from the study only if they were unable to read the forms required for the study due to mental incapacity or language barrier. The study evaluated preoperative psychological distress and presurgical substance abuse to determine the effects of these variables on the duration of opioid use postoperatively. The study defined “prolonged opioid use” as opioid treatment lasting for more than six months. All data analysis were controlled for which surgery the patient received [21].

Carroll et al. discovered that the use of prescribed opioid preoperatively, symptoms suggestive of depressive disorders, and high self-perceived addiction risk were each independently associated with prolonged opioid use. Patients who used opioid medications prescribed by medical professionals in the preoperative period were more likely to use their postoperative opioids for a longer period of time (73% longer [95% CI 41%– 87%], P=0.0009), often until their pain had almost completely subsided. Patients who ranked themselves as high risk when asked via survey, “How likely do you think it is that you will develop an addiction problem from pain medication you take after surgery?” were also at increased risk during their postoperative period. Every 1-point increase (on a 4-point scale, with higher scores indicating increased self-perceived risk) was independently associated with a 53% increase (95% CI 23%–71%; P=0.003) in the duration of opioid use postsurgically. Self-perceived risk of addiction was an even stronger predictor of addiction than some traditionally recognized risk factors including a family history of substance use disorder, history of illicit substance abuse, and tobacco and/or alcohol use. The study also found that increased depressive symptoms (as measured by Beck Depression Inventory scores) were correlated with greater risk of prolonged opioid use (each 10-point increase in the 63-point score was related to a 42% increase (95% CI 18%–58%; P=0.002) in the length of opioid use). In each case, the variable’s predictive value was lessened by the addition of pain as a predictor variable. When measures of pain were included (pain duration and severity), they demonstrated only small effects on time to opioid cessation that were not statistically significant. The findings of this study suggest that delays in the path to opioid cessation cannot be explained by pain duration alone and is instead better foretold by patient level predictors (here: preoperative opioid use, self-perceived risk of addiction, and depressive symptoms) [21]. This study was limited by the small sample size, as well as the fact that it too lists only correlations without any attempt to explain causation. Furthermore, it failed to account for patients receiving revision surgeries who may require prolonged opioid regimens due to subsequent procedures sometimes weeks after the initial surgery. Additionally, this data may not be applicable outside of the original study population.

Sun et al. intended to characterize the risk factors for chronic opioid use among patients who were opioid naive. In addition, they sought to define individual risk factors for chronic opioid use in surgical patients as compared to non-surgical patients. Their chart review drew its samples from administrative health claims, both inpatient and outpatient, dated between January 1, 2001 and December 31, 2013 as provided by MarketScan (Truven Health Analytics). This allowed Sun et al. to include 641,941 opioid-naive surgical patients (169,666 men; mean age 44.0, SD=12.8 years), and 18,011,137 opioid-naive nonsurgical patients (8,849,107 men; mean age 42.4, SD=2.6 years). Data were assembled via the inclusion of specific diagnostic codes (International Classification of Diseases, Ninth Revision (ICD-9)), procedure codes (Current Procedural Terminology (CPT)), and respective dates from encounters. The pharmacy data included fill date, quantity supplied, number of days supplied, and the National Drug Code which was linked to Red Book data (Truven Health Analytics) to obtain the generic name and dose of the prescribed drug [20].

The included surgeries were TKA, total hip arthroplasty, laparoscopic cholecystectomy, open cholecystectomy, laparoscopic appendectomy, open appendectomy, cesarean delivery, FESS, cataract surgery, TURP, and simple mastectomy. Patients included in this study were 18 to 64 years old and were continuously enrolled in Market Scan service for at least three calendar years, including the year before and the year after the procedure. Patients who underwent two or more of the 11 studied surgical procedures were excluded. Opioid-naive (control) patients were defined as those who did not fill an opioid prescription for 12 months prior to their procedure. Medications included in the study were fentanyl (patch or oral form), hydrocodone, hydromorphone (oral form), methadone, morphine, oxymorphone, and oxycodone. Prescriptions containing hydrocodone in cough/cold formulation and analgesic preparations containing codeine, such as acetaminophen/codeine were excluded. Using ICD-9 codes, the authors of this paper used multivariable logistic regression to determine odds ratios for chronic opioid use associated with each procedure, controlling for age, sex, and preoperative use of medications to account for the differences in the surgical and nonsurgical groups [20].

The authors reported the incidence of chronic opioid use was significantly higher for all procedures investigated except for TURP, laparoscopic appendectomy, FESS, and cataract surgery. Odds ratios ranged from 1.28 (95%CI, 1.12-1.46) to 5.10 (95%CI, 4.67-5.58) for cesarean section and TKA, respectively. However, the most important risk factor for chronic opioid use postsurgically was drug abuse history with an odds ratio of 3.15 (95% CI 2.24-4.40, P<0.001). After drug abuse history, alcohol abuse history (OR 1.83, 95% CI 1.35-2.47, p<0.001), preoperative benzodiazepine use (OR 1.82, 95% CI 1.63-2.04, p<0.001), and preoperative antidepressant therapy (OR 1.65, 95% CI 1.47-1.84, p<0.001) were the next most implicated. The next most important risk factor was age more than 50 years (OR 1.74, 95% CI 1.56-1.93, p<0.001), followed by male sex (OR 1.34, 95% CI 1.22-1.47, p<0.001), and history of depression (OR 1.15, 95% CI 1.02-1.30, p=0.03). Sun et al. concluded that the most important risk factors for postsurgical chronic opioid use relate to presurgical use of drugs, particularly opioids, benzodiazepines, and antidepressants. After these risk factors, demographic features take priority, with age more than 50 years and male sex increasing risk. Finally, the psychiatric diagnosis of depression is the weakest of the statistically significant risk factors they found. Sun et al. called for further research to discover whether their findings apply to other surgical procedures and populations, along with research for the development of interventions during the postoperative period which could reduce risk of chronic opioid use [20].

The purpose of this chart review was to obtain disease-oriented evidence of risk for developing chronic opioid use with certain presurgical risk factors and with certain surgeries. Sun et al. managed to obtain a massive sample size allowing for excellent statistical power. The article also clearly defined specifically which opioid medications were relevant in the context of postsurgical pain management and focused on surgeries with variable degrees of trauma involved with analysis for confounding variables. It may be important to note that the authors were unable to effectively represent all socioeconomic levels as all the patients included in their dataset were privately insured [20].

In Brummett's study titled “New Persistent Opioid Use After Minor and Major Surgical Procedures in US Adults,” a retrospective approach was used to study the incidence of new persistent opioid use after minor and major surgical procedures. The Clinformatics Data Mart, nationwide insurance claims data from 2013 to 2014 was gathered and used to identify patients aged 18 to 64 who had major or minor surgery without opioid use during the year prior to surgery. Thirteen common elective surgeries were targeted as selection criteria and separated into major and minor categories for the purpose of this study. This sample was then control-matched to a 10% cohort of the 492,177 patients’ data analyzed that did not have surgery or use of opioids within one year of a chosen date. These patients were then assessed for opioid use of more than 90 days after surgery and their risk factors for persistent opioid use were evaluated. A sample population of 36,177 was identified [10].

The primary outcome studied was new persistent opioid use, defined as an opioid prescription filled between 90 and 180 days after the surgical procedure. The study found that the average initial surgical opioid prescription contained between 30-45 tablets. Tobacco use, alcohol use, substance abuse disorders, and comorbid medical conditions (such as preoperative pain disorders, anxiety, and mood disorders) were identified as risk factors of persistent opioid use. Another finding of note was that the risk of new persistent opioid use was similar between the major and minor surgery groups, with those using opioids within 30 days prior to surgery being twice as likely to have persistent postsurgical use. Risk factors discovered to be independently associated with continued postoperative opioid use were anxiety, depression, back pain, neck pain, arthritis, and centralized pain conditions [10].

According to the findings of this study and the average amount of annual outpatient surgical procedures, the authors project that approximately two million new patients will develop persistent opioid use (as defined by this study) after their procedure every year. Persistent opioid use was found as an important and under-diagnosed complication resultant of desires to decrease morbidity and mortality of surgical procedures. This study has a high statistical power due to the extensive reporting of data and statistical analysis coupled to a large sample size. The limitations of this article include the lack of specific definitions regarding the nuances of persistent use versus abuse/misuse disorders. This blurs the line between possible appropriate opioid prescriptions and risk factors for abuse disorders. This study further supports the growing data suggesting there are patient-centered risk factors for persistent postsurgical opioid abuse, but the type of surgical procedure and amount of pain experienced during the procedure do not appear to be statistically significant variables with persistent opioid use [10].

Clarke et al. conducted a retrospective cohort study to describe rates and risk factors for prolonged use of opioids postoperatively in opioid-naive patients after major elective surgery. The study enrolled 39,140 opioid-naive patients age 66 or older who had a major elective surgery, with the intent to measure opioid use after discharge using data from the Canadian Institute for Health Information, the Ontario Health Insurance Plan database, Registered Persons database, and the Ontario Drug Benefit database. Prolonged opioid use was defined as ongoing outpatient prescriptions for opioids in excess of 90 days after surgery. Patients were included if they underwent one of nine pre-selected, elective, major surgeries during the seven year study period. The surgeries of interest were chosen due to the variety in the level of trauma and location. The study included isolated coronary artery bypass graft through sternotomy, open (thoracotomy) lung resection, and lung resection using video assisted thoracoscopic surgery (amongst many others). Patients with pre-existing pain disorders before their surgeries and those who required palliative care were excluded [18].

The data collected allowed the authors to categorize their participants in cohorts for characteristics, socioeconomic status, residence, comorbidities, preoperative drug use, and survival. Using multivariable logistic regression modelling, the authors adjusted for associations of age (66-75, 76-85, >86), sex, surgery, fifth of neighborhood income, rural residence, comorbidities (specifically coronary artery disease, heart failure, cerebrovascular disease, peripheral vascular disease, diabetes, hypertension, pulmonary disease, chronic renal insufficiency, malignancy), and preoperative drug use (specifically β blockers, angiotensin-converting enzyme (ACE) inhibitors, angiotensin receptor blockers, statins, benzodiazepines, selective serotonin reuptake inhibitors (SSRIs)) with prolonged opioid use after discharge [18].

Of the 39,140 patients who had not previously used opioids and underwent major elective surgery, 49.2% (n=19,256) used opioids in the early post-discharge period and 3.1% (n=1,229) used opioids for a prolonged period after discharge. In contrast to other studies cited, the type of surgical procedure was a significant determining factor for prolonged postoperative opioid use. Clarke et al. found that patients who had undergone open (8.5%) or minimally-invasive intrathoracic procedures (6.3%) were at the highest risk for prolonged opioid use after their surgeries. When compared to radical open prostatectomies, open and minimally invasive surgeries of the thorax were associated with higher risk for prolonged opioid use postoperatively (OR 2.58, 95% CI 2.03 to 3.28; OR 1.95, 95% CI 1.36 to 2.78, respectively). After the author’s multivariable risk adjustment, the patient characteristics of younger age, lower fifth of neighborhood income, diabetes, heart failure, and pulmonary disease, use of benzodiazepines, SSRIs, and ACE inhibitors before surgery were found to be associated with significantly increased risk of prolonged opioid use [18].

Clarke et al. conducted a study of significant statistical power due to its large sample size whose individual subjects were chosen with very specific inclusion and exclusion criteria. The statistical analyses used and the data yielded were transparently displayed both pre- and post-adjustment. This article is also one of the few that took into account patient socioeconomic status, although it did so imperfectly by assuming socioeconomic status by patients’ neighborhoods. Similar to other studies that review patient data through healthcare claims, there is no way to control for clerical errors. Otherwise, this study did not investigate mental health diagnoses specifically but rather used corresponding medications, meaning those results are not specific for each diagnosis. Finally, because each participant was over 65 years of age, there was little heterogeneity in the sample.

Finally, a study by Katz et al. aimed to determine the mental and physical health predictors of incident nonmedical prescription opioid use (NMPOU) and abuse/dependence and the impact of comorbidity in a longitudinal, nationally representative sample using the results of the National Epidemiologic Survey on Alcohol and Related Conditions (NESARC), conducted by the National Institute on Alcohol Abuse and Alcoholism. This gave access to results from over 34,653 participants who completed both wave one of the survey in 2001-2002 and wave two in 2004-2005. The inclusion criteria were set by the original surveyor, non-military, non-institutionalized individuals, aged 18 years and older, and living within the United States for both waves one and two of the survey. The exclusion criteria of the survey were imposed at wave two for any participants that were deceased, deported, physically or mentally ill, or on active military duty. Furthermore, the researchers excluded any participants who were found to meet the qualifications of lifetime abuse/dependence during the wave one survey, when assessed by the Alcohol Use Disorder and Associated Disabilities Interview Schedule-IV (AUDADIS-IV) questionnaire [23].

The data gathered from the NESARC were weighted to reflect the national civilian population based on the 2000 census. The Alcohol Use Disorder and AUDADIS-IV questionnaires were utilized to assess for psychiatric disorders. Physical conditions were based on self-reports of physician-diagnoses. During statistical analysis for the significance of each individual risk factor, a total of 12 different logistic regression models were implemented to control for confounders and other important risk factors [23].

The four major outcomes being evaluated in this article are (1) the relationship between sociodemographic information at wave one and its association to incident opioid NMPOU and abuse/dependence in wave two, (2) mental disorder variables at wave one and their association to incident NMPOU and abuse/dependence at wave two, (3) physical condition variables at wave one and their association to incident NMPOU and abuse/dependence at wave two, (4) the additive effects of variables from wave one and their association to incident NMPOU and abuse/dependence at wave two [23].

Katz et al. were able to draw many significant conclusions from the analysis of risk factors in this nationally representative survey, the first of which was the significance of several chronic physical conditions (i.e., arteriosclerosis or hypertension, cardiovascular disease, arthritis, and any assessed medical condition) as a positive risk factor for the onset of opioid abuse/dependence. This remained true even after controlling for sociodemographic factors and Axis I and II mental disorders, but not for the prediction of NMPOU risk. The article was also able to display a dose-response relationship of Axis I and II disorders and physical conditions in predicting incident prescription opioid abuse/dependence. Mental disorders were also found to be a significant risk factor for both NMPOU and abuse/dependence, even after controlling for comorbid mental disorders and physical conditions. Comorbidity of mental disorder is a significant risk factor for NMPOU and abuse/dependence, which, when combined with other less-impactful risk factors, can lead to significantly increased patient risk. For instance, patients with comorbid chronic pain and mental disorders receive more opioid prescription at higher doses than those with chronic pain alone [23].

In terms of clinical significance this paper continues to be relevant despite its inability to suggest causation. The first wave of this survey was conducted during the early stages of the opioid epidemic and the second wave was collected during the subsequent escalation of the opioid epidemic. Though this may have slightly inflated the incidence of new NMPOU and abuse/dependence relative to its current state, the authors of this review believe that taken at face value the risk factors studied have remain aligned with current works. Furthermore, while this study has made the inference that NMPOU can be a significant risk factor for developing an OUD, it could not quantify the severity of NMPOU required to cause a patient to develop OUD [23]. Although this study is not focused on the postoperative population, the works of Brummett and Carroll suggest that the differences between surgical and non-surgical populations are found to be negligible compared to patient-centered risk factors for OUD [10, 21].

The large sample size and the proven validity of the NESARC survey suggest this study may be reproducible in other samples of the population. The authors’ transparency with respect to statistical analysis and control for confounding variables are further strengths. The criticisms discussed above, including age of the study and the period of the opioid crisis during which it was executed, are limitations. All of these points considered, this article draws important conclusions and agrees with the current body of literature on many important risk factors for developing an OUD, making it applicable in today’s opioid crisis climate [23].

A summary of findings from these six articles is available within this text (Table 2). The authors of this review suggest that history of substance abuse, any physical malady, mental health history, and use of sedatives/hypnotics be given great weight in identifying patients at greater risk of developing an OUD. These risk factors were listed in at least three of the six articles reviewed. The authors also suggest the use of any age as a risk factor for OUD be discontinued as virtually all the sources cited in this review disagreed. Furthermore, this review should not be used to predict at risk age groups as some of the articles used age as an inclusion/exclusion criteria.

**Table 2:** Predictors of OUD OUD - opioid use disorder, SSRI - selective serotonin reuptake inhibitors, ACE - angiotensin-converting enzyme.

Major Risk Factor	Examples (if applicable)	Number of Articles Cited	Citation Numbers
History of substance abuse	“any history of substance abuse disorder,” “History of illicit substance abuse,” or “History of illegal drug use or prescription drug abuse.”	4	[20] [21] [22] [23]
Any physical maladies	“headache,” “neck pain,” “central pain syndromes” “arthritis,” “back pain,” “diabetes,” and “pulmonary disease”	4	[10] [17] [22] [23]
Mental health history	“axis 1 and 2 disorders” including “anxiety” and “depression”	3	[10] [22] [23]
Sedative/Hypnotic use	“benzodiazepines”	3	[18] [20] [22]
Depression	“depression” including “symptoms of…”	3	[10] [20] [21]
Younger age*	“young age” “18-30 years”	2	[22] [23]
Older age*	“65+ years”, “50+ years”	2	[20] [22]
Alcohol abuse history	“alcohol abuse”	2	[20] [21]
Anti-depressant use	“Anti-depressant” “SSRI”	2	[18] [20]
Male gender	“male”	2	[20] [23]
Single	“unmarried”	1	[23]
Race	“white, non-Hispanic”	1	[23]
Comorbidity of mental health and physical condition	Ex. Depression and back pain	1	[23]
High intensity opioid therapy	“Higher opioid dose,” “Increased length of opioid use,” “use of Schedule II opioids”	1	[22]
Preoperative opioid use		1	[21]
High self-assessed risk of addiction		1	[21]
Tobacco use		1	[21]
Family history of substance abuse		1	[21]
Low socioeconomic status	“Lower fifth of neighborhood income”	1	[18]
Use of ACE-inhibitors		1	[18]
*The authors suggest the removal of any age as a risk factor for OUDs as no consensus has been reached and virtually all the sources cited in this review disagreed. Furthermore, this review should not be used to predict at-risk age groups as some of the articles used age as an inclusion/exclusion criteria.			

This review is not without its limitations. This paper is a literature review, without any experimental process performed, the authors could only assess the merits of each article from the data provided by each publication. The nature of the individual studies described in this article also allows only for a conclusion of correlation without insight into causative relationships. Furthermore, only six total studies were identified meeting our inclusion criteria and more data may lead to stronger conclusions. These studies were fairly heterogenous in terms of variables considered and the study's definition of OUD, perhaps leading to technical issues in meta-analysis.

The authors of this article hope that information regarding the risk factors for OUDs will be useful in the development of a preoperative screening tool and tiered opioid pain management plan. Altogether, the aforementioned shortcomings prevented the authors of this review from conducting a stronger evidence-based meta-analysis study protocol. There is a need for more uniform studies involving the use of tools that predict risk of OUD and the development of such tools. In this context, evaluating physical characteristics of pain and percentage of patients still using pain medications for an extended period of time postoperatively when compared to current standards for pain management would elucidate if preoperative screening is effective. Only then can knowledge of such risk factors be used to encourage the vigilance of medical providers, allowing for the development of a heightened index of suspicion given the correct clinical picture.

## Conclusions

This review has found the following are useful predictors of the risk of OUD in the adult postsurgical population: history of substance abuse, any physical malady, mental health history, and use of sedatives/hypnotics. The validity of these findings is limited by heterogeneity between studies, limited number of studies meeting inclusion criteria, and lack of data suggesting a causative relationship. It is the authors’ conclusion that identifying certain risk factors could allow for the development of a heightened index of suspicion therefore encouraging the vigilance of medical providers when prescribing opioids. This vigilance is a critical component of turning the tide of the opioid epidemic.
